# Sex hormone-related neurosteroids differentially rescue bioenergetic deficits induced by amyloid-β or hyperphosphorylated tau protein

**DOI:** 10.1007/s00018-015-1988-x

**Published:** 2015-07-22

**Authors:** Amandine Grimm, Emily E. Biliouris, Undine E. Lang, Jürgen Götz, Ayikoe Guy Mensah-Nyagan, Anne Eckert

**Affiliations:** Neurobiology Laboratory for Brain Aging and Mental Health, Transfaculty Research Platform, Molecular and Cognitive Neuroscience, University of Basel, Wilhelm Klein-Str. 27, 4012 Basel, Switzerland; Psychiatric University Clinics, University of Basel, Wilhelm Klein-Str. 27, 4012 Basel, Switzerland; Biopathologie de la Myéline, Neuroprotection et Stratégies Thérapeutiques, INSERM U1119, Fédération de Médecine Translationnelle de Strasbourg (FMTS), Université de Strasbourg, Bâtiment 3 de la Faculté de Médecine, 11 rue Humann, 67 000 Strasbourg, France; Clem Jones Centre for Ageing Dementia Research (CJCADR), Queensland Brain Institute (QBI), The University of Queensland, Brisbane, 4072 QLD Australia

**Keywords:** Mitochondria, Neurosteroids, Bioenergetics, Amyloid-β peptide, Tau protein

## Abstract

Alzheimer’s disease (AD) is an age-related neurodegenerative disease marked by a progressive cognitive decline. Metabolic impairments are common hallmarks of AD, and amyloid-β (Aβ) peptide and hyperphosphorylated tau protein—the two foremost histopathological signs of AD—have been implicated in mitochondrial dysfunction. Neurosteroids have recently shown promise in alleviating cognitive and neuronal sequelae of AD. The present study evaluates the impact of neurosteroids belonging to the sex hormone family (progesterone, estradiol, estrone, testosterone, 3α-androstanediol) on mitochondrial dysfunction in cellular models of AD: human neuroblastoma cells (SH-SY5Y) stably transfected with constructs encoding (1) the human amyloid precursor protein (APP) resulting in overexpression of APP and Aβ, (2) wild-type tau (wtTau), and (3) mutant tau (P301L), that induces abnormal tau hyperphosphorylation. We show that while APP and P301L cells both display a drop in ATP levels, they present distinct mitochondrial impairments with regard to their bioenergetic profiles. The P301L cells presented a decreased maximal respiration and spare respiratory capacity, while APP cells exhibited, in addition, a decrease in basal respiration, ATP turnover, and glycolytic reserve. All neurosteroids showed beneficial effects on ATP production and mitochondrial membrane potential in APP/Aβ overexpressing cells while only progesterone and estradiol increased ATP levels in mutant tau cells. Of note, testosterone was more efficient in alleviating Aβ-induced mitochondrial deficits, while progesterone and estrogen were the most effective neurosteroids in our model of AD-related tauopathy. Our findings lend further support to the neuroprotective effects of neurosteroids in AD and may open new avenues for the development of gender-specific therapeutic approaches in AD.

**Electronic supplementary material:**

The online version of this article (doi:10.1007/s00018-015-1988-x) contains supplementary material, which is available to authorized users.

## Introduction

Alzheimer’s disease (AD) is an age-related neurodegenerative disease that accounts for more than 60 % of all dementia cases. AD will become increasingly burdensome and costly in the coming years as its prevalence is expected to double within the next two decades [1]. The disease is characterized by cognitive deficits and memory loss and, from a histopathological point of view, by the presence of amyloid-β (Aβ) plaques and neurofibrillary tangles (NFTs) composed of abnormally hyperphosphorylated tau protein in the brain. Genetic studies link mutations in the amyloid-β protein precursor (APP) to familial AD (FAD) cases. These mutations lead to an increased Aβ production in the brain of AD patients [2]. Interestingly, no mutations in the tau encoding gene have been identified so far in FAD. However, such mutations were detected in familial frontotemporal dementia with Parkinsonism linked to chromosome 17 (FTDP-17) leading to NFT formation [3]. Both histopathological hallmarks of AD, Aβ, and abnormal tau protein induce synaptic disintegration and oxidative stress, and disrupt Ca^2+^ homeostasis leading to neurodegeneration [4–6]. Even if FAD cases only represent a small percentage of all AD patients (less than 1 %), the vast majority of non-familial AD, termed sporadic AD (SAD), and FAD patients have a number of disease features in common, especially the presence of mitochondrial deficits already at an early AD stage [7–10].

Indeed, mitochondria are essential components of metabolic function and serve as the “powerhouses” of cells, providing energy in the form of adenosine triphosphate (ATP) that is used by cells to power a variety of cellular processes including apoptosis, intracellular calcium homeostasis, alteration of the cellular reduction–oxidation (redox) state, and synaptic plasticity [11, 12]. Mounting evidence suggests that mitochondrial dysfunction serves as a catalyst in AD, since the disease is associated with a decline in bioenergetic activity and an increase in oxidative stress that can already be detected at early disease stage in both FAD and SAD [10, 13–15]. Indeed, brain glucose hypometabolism has been observed in AD patients well before the onset of clinical symptoms [16]. This disease characteristic was also observed in AD mouse models, bearing mutations in APP and/or tau protein, in which mitochondrial dysfunction is already obvious before the appearance of Aβ deposits, NFT formation, and cognitive impairments (reviewed in [10]). With regard to their critical role in the early pathogenesis of both SAD and FAD, mitochondria therefore represent an interesting target for the development of novel treatment avenues.

Based on recent discoveries, interventions that target mitochondrial deficits, such as the use of neurosteroids, may serve as potential strategies for the treatment of AD [17–19]. In 1981, Baulieu and colleagues characterized a new category of steroids that is synthesized in the nervous system and persists in substantial amounts after removal of the peripheral steroidogenic glands [20]. This category of molecules was dubbed “neurosteroids.” In a recent study, we characterized the bioenergetic modulating profile of seven structurally diverse neurosteroids that are known to be involved in the regulation of brain functions [21–25], namely progesterone, estradiol, estrone, testosterone, 3α-androstanediol, DHEA, and allopregnanolone. We had found that most of the steroids tested by us improved the bioenergetic activity in neuronal cells by increasing ATP levels, the mitochondrial membrane potential (MMP), and mitochondrial respiration, in a similar pattern in SH-SY5Y neuroblastoma cells as well as in primary neuronal culture [19]. Our results provided new insights in re-defining the biological model of how neurosteroids control neuronal functions, further emphasizing the role of neurosteroids in neuroprotection.

In line with that study, a growing body of evidence attests to the neuroprotective effects of neurosteroids, especially estrogenic compounds, against AD-related cellular injury (reviewed in [18]). However, little is known about the influence of neurosteroids on AD-related mitochondrial dysfunction. Additionally, the primary focus of neurosteroid treatment in AD has centered in the past on Aβ plaques rather than tau-related NFTs.

Thus, the aim of the current study was to assess whether neurosteroids of the sex hormone family can attenuate the toxic effects of Aβ and/or abnormal tau on mitochondria and whether the influence of neurosteroids on tau-related deficits independent of Aβ can be differentiated. For this purpose, we investigated the effects of progesterone, estradiol, estrone, testosterone, and 3α-androstanediol on bioenergetics in SH-SY5Y neuroblastoma cells stably transfected with wild-type human APP (APP cells) or the empty vector (Mock cells), and wild-type human tau (wtTau cells) or mutated tau (P301L cells), respectively. Of note, both AD cell culture models, APP and P301L cells, express a variety of neuronal receptors, including steroid receptors (progesterone, estrogen, and androgen receptors) and exhibit the characteristics of a mitochondrial malfunction when compared to their respective controls [26–29]. They shared decreased ATP levels as well as impaired mitochondrial respiration [26, 27] but differed in the underlying mechanisms. Thus, APP cells presented a defect in complex IV activity [26], whereas complex I activity was impacted by mutant tau in P301L cells [27]. On the basis of our previous findings, it was therefore of interest to examine whether neurosteroids are able to alleviate mitochondrial deficits manifested in these AD cellular models. In particular, their impact on ATP production, mitochondrial membrane potential (MMP), mitochondrial respiration, and glycolysis was investigated.

## Materials and methods

### Chemicals and reagents

Dulbecco’s-modified Eagle medium (DMEM), fetal calf serum (FCS), penicillin/streptomycin, progesterone, 17β-estradiol, estrone, 3α-androstanediol, pyruvate, hydrogen peroxide (H_2_O_2_), retinoic acid, and brain-derived neurotrophic factor (BDNF) were from Sigma-Aldrich (St. Louis, MO, USA). Glutamax, B27 supplement, and neurobasal medium were from Gibco Invitrogen (Waltham, MA, USA). XF Cell Mitostress kit was from Seahorse Bioscience (North Billerica, MA, USA). Testosterone was from AppliChem (Darmstadt, Germany). Horse serum (HS) was from Amimed, Bioconcept (Allschwil, Switzerland).

### Cell culture

In the present study, human neuroblastoma SH-SY5Y cells stably expressing vector alone (pCEP4, control cells: Mock) or the entire coding region of human wild-type APP (APP695, APP cells) were used as described previously [30]. Stably transfected cell clones were selected with hygromycin. Cell cultures were kept under steady selection pressure and checked on a routine basis for APP expression levels [26]. APP cells secreted Aβ levels in the pg/mL range (around 150 pg/mL Aβ1-40 compared to approximately 50 pg/mL secreted by vector control cells) [26, 31]. In addition, human SH-SY5Y neuroblastoma cells stably transfected with wild-type (wtTau cells) and P301L mutant tau (P301L cells) were used [32]. SH-SY5Y cells, either harboring expression constructs encoding the longest 4-repeat isoform of human tau (wtTau) or tau with the pathogenic FTDP-17 mutation P301L (P301L), showed similar tau expression levels [27, 32], but the presence of the P301L mutation is required for abnormal tau hyperphosphorylation and filament formation [32]. Cells were kept under steady selection pressure with G418 (125 μg/ml) and screened on a regular basis by routine histochemical and biochemical assays.

Cells were grown at 37 °C in a humidified incubator chamber under an atmosphere of 7.5 % CO_2_ in DMEM supplemented with 10 % (v/v) heat-inactivated FCS, 5 % (v/v) heat-inactivated HS, 2 mM Glutamax, and 1 % (v/v) penicillin/streptomycin. Cells were passaged 1–2 times per week (the maximum number of passages after taking the cells into culture did not exceed 15) and plated for treatment when they reached 80–90 % confluence.

In experiments using differentiated cells, 96-well plates were coated with 0.05 mg/ml collagen. Mock and APP cells were plated at a density of 5 × 10^4^ cells/well, and wtTau and P301L cells were plated at a density of 1 × 10^4^ cells/well. Cells were grown in Neurobasal medium supplemented with 2 % B27, 2 mM Glutamax, and 1 % (v/v) penicillin/streptomycin. Cells were treated for 5 days with 10 μM of retinoic acid and 50 ng/ml of BDNF (brain-derived neurotrophic factor) before the treatment with steroids started.

### Treatment paradigm

Assessment of cell viability was performed on SH-SY5Y neuroblastoma cells (Mock, APP, wtTau and P301L cells) to determine the potential toxic concentration range of neurosteroids (from 10 to 1000 nM, data not shown) using an MTT reduction assay (Roche, Basel, Switzerland). On the basis of the MTT results, and according to previous data obtained in untransfected SH-SY5Y neuroblastoma cells [19], a concentration of 100 nM was then selected and used in all assays. Cells were treated 1 day after plating for 24 h either with DMEM (untreated control condition) or with a final concentration of 100 nM progesterone, 17β-estradiol, estrone, testosterone, or 3α-androstanediol made from a stock solution in DMSO (final concentration of DMSO <0.002 %, no effect of the vehicle solution (DMSO) alone compared to the untreated condition). To limit cell growth and to force the cells to function in an aerobic state (high oxidative phosphorylation), the treatment medium contained only a low amount of fetal calf serum (5 % FCS) as well as glucose (1 g/l) and was supplemented with 4 mM pyruvate.

For the stress experiments, cells were first pre-treated for 24 h with progesterone, estradiol, and testosterone and then treated for 3 h with H_2_O_2_ (250 μM for APP cells and 500 μM for Mock and P301L cell). Then ATP assays were performed and repeated at least 3 times.

### ATP levels

Total ATP content was determined using a bioluminescence assay (ViaLighTM HT; Cambrex Bio Science) according to the instructions of the manufacturer, as previously described [26]. Cells were plated in 5 replicates into a white 96-well cell culture plate at a density of 1.5 × 10^4^ cells/well. The bioluminescent method measures the formation of light from ATP and luciferin by luciferase. The emitted light was linearly related to the ATP concentration and was measured using multilabel plate reader VictorX5 (Perkin Elmer).

### Determination of mitochondrial membrane potential

The MMP was measured using the fluorescent dye tetramethylrhodamine, methyl ester, perchlorate (TMRM) [19]. Cells were plated in 6 replicates into a black 96-well cell culture plate at a density of 1.5 × 10^4^ cells/well. Cells were loaded with the dye at a concentration of 0.4 μM for 15 min. After washing twice with HBSS, the fluorescence was detected using the multilabel plate reader VictorX5 (PerkinElmer) at 531 nm (excitation)/595 nm (emission). Transmembrane distribution of the dye is dependent on MMP.

### Mitochondrial respiration

The investigation of mitochondrial respiration and cellular glycolysis was performed using the Seahorse Bioscience XF24 analyser. XF24 cell culture microplates were coated with 0.1 % gelatin and cells were plated at a density of 2.5 × 10^4^ cells/well in 100 μl treatment medium containing 5 % FCS, 1 g/l glucose, and 4 mM pyruvate. After neurosteroid treatment, cells were washed with 1X pre-warmed PBS and 500 μl of DMEM containing 1 g/l of glucose and 4 mM of pyruvate were added in each well. The oxygen consumption rate (OCR) and extracellular acidification rate (ECAR) were recorded simultaneously before and after the sequential injection of (1) oligomycin (0.5 μM), (2) FCCP (2 μM), and (3) antimycin A and rotenone (0.5 μM and 1 μM respectively). Data were extracted from the Seahorse XF-24 software, and bioenergetic parameters (basal respiration, ATP turnover, maximal respiration, spare respiratory capacity, and glycolytic reserve) were calculated according to the guideline of the company.

### Statistical analysis

Data are given as the mean ± S.E.M. Statistical analyses were performed using Graph Pad Prism software (version 5.02 for Windows, San Diego, California, USA). For statistical comparisons of more than two groups, one-way ANOVA was used, followed by a Dunnett’s multiple comparison tests versus the control. For statistical comparisons of two groups, student unpaired *t* tests were used. *P* values <0.05 were considered statistically significant.

## Results

### APP and hyperphosphorylated tau differentially impair mitochondrial bioenergetics

**Fig. 1:**
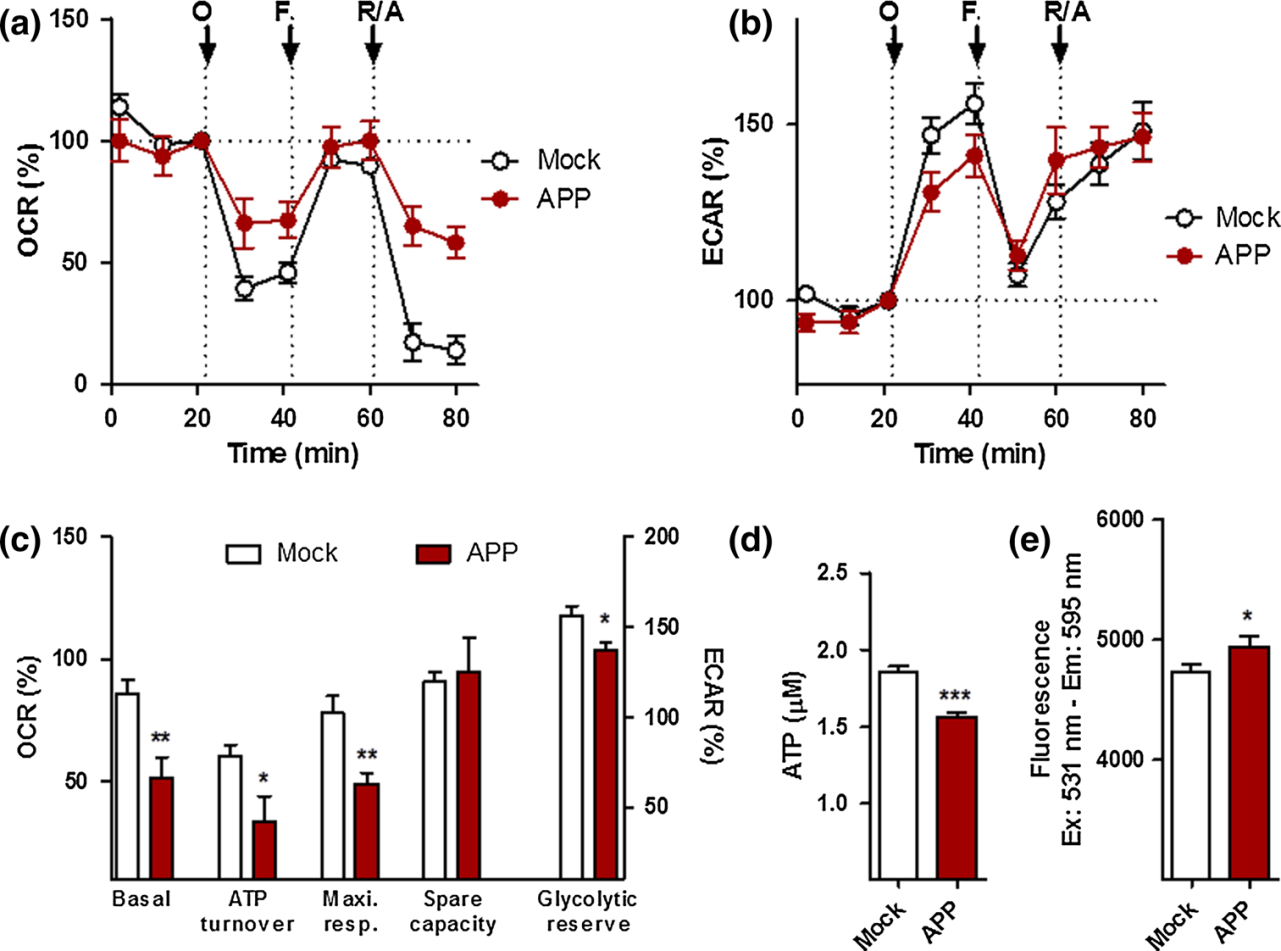
Characterization of bioenergetic deficits in APP cells. **a** Oxygen consumption rate (OCR) and **b** extracellular acidification rate (ECAR) of Mock and APP cells were simultaneously measured using a XF24 Analyzer (Seahorse Bioscience). The sequential injection of mitochondrial inhibitors is indicated by *arrows* (see details in the “Materials and methods” section). Changes in the OCR and ECAR are shown as a percent change from baseline (=100 %, dashed line). **c** Values corresponding to the different bioenergetic parameters are represented as mean ± SEM (*n* = 8–10 replicates). **d** ATP levels and **e** mitochondrial membrane potential (MMP) in Mock and APP cells. Values represent the mean ± SEM (*n* = 12–18 replicates of three independent experiments). Student unpaired *t* test, *P < 0.05; ***P < 0.001. *O* oligomycin, *F* FCCP, *R/A* rotenone/antimycin A

To measure the efficiency of mitochondrial respiration and cellular bioenergetics in APP/Aβ overexpressing cells, we simultaneously monitored in real time the oxygen consumption rate (OCR) (Fig. 1a), an indicator of mitochondrial respiration, as well as the extracellular acidification rate (ECAR) (Fig. 1b), an indicator of glycolysis, using a Seahorse Bioscience XF24 Analyzer. We first performed experiments on untreated control and APP cells to record AD-related differences in OCR and ECAR readouts. A significant decrease (about 50 %) in basal respiration, ATP turnover, maximal respiration, as well as glycolytic reserve was observed in APP cells when compared to control cells (Fig. 1c), paralleled by a reduction in ATP levels (−20 % compared to control cells) (Fig. 1d). Surprisingly, a slight increase in MMP was observed in APP cells (Fig. 1e), translating to a hyperpolarization of the mitochondrial membrane potential.

**Fig. 2:**
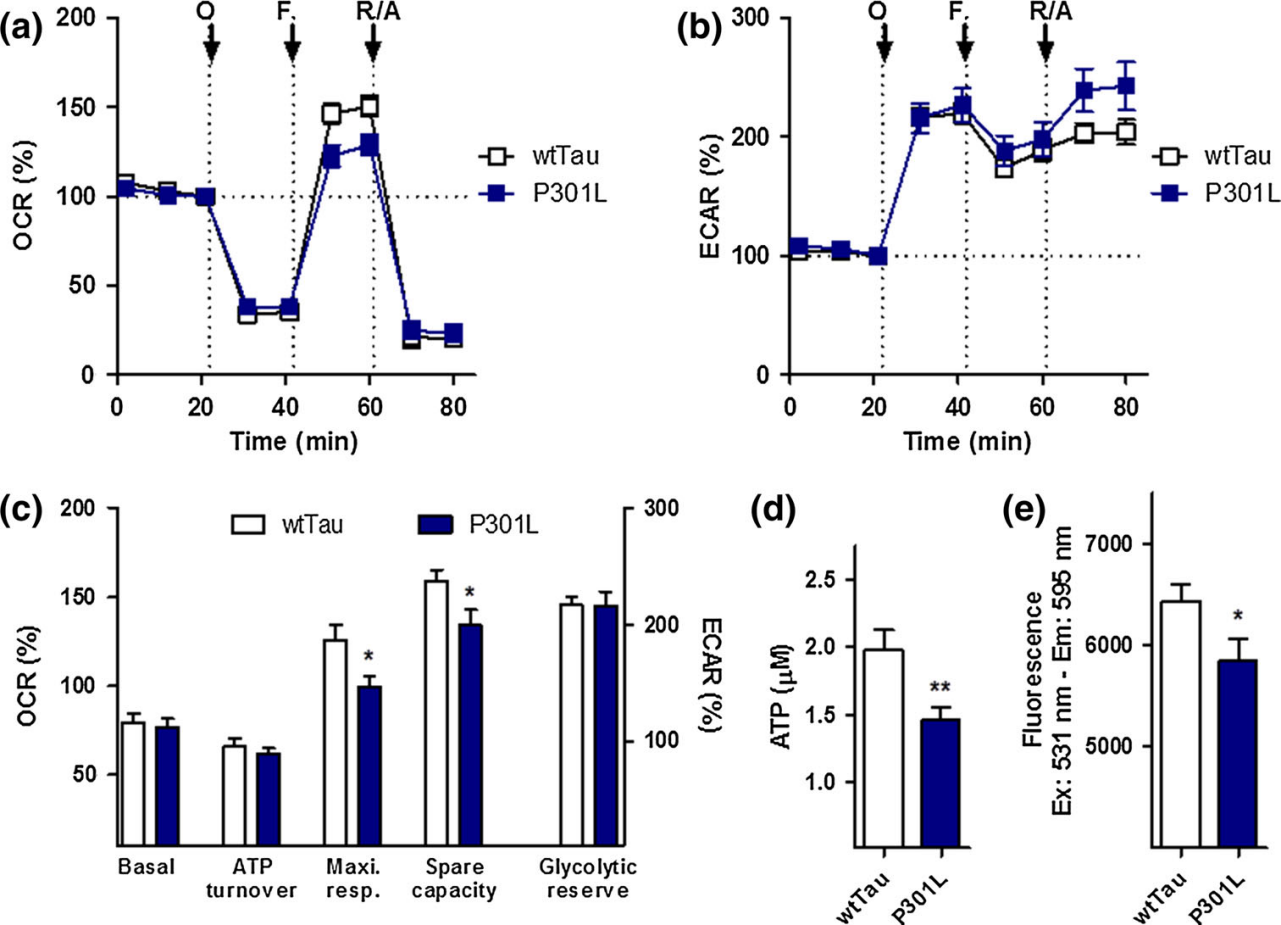
Characterization of bioenergetic deficits in P301L cells. **a** Oxygen consumption rate (OCR) and **b** extracellular acidification rate (ECAR) of wtTau and P301L cells were simultaneously measured using a XF24 Analyzer (Seahorse Bioscience). The sequential injection of mitochondrial inhibitors is indicated by arrows (see details in the “Materials and methods” section). Changes in the OCR and ECAR are shown as a percent change from baseline (=100 %, *dashed line*). **c** Values corresponding to the different bioenergetic parameters are represented as mean ± SEM (*n* = 8–10 replicates). **d** ATP levels and **e** mitochondrial membrane potential (MMP) in wtTau and P301L cells. Values represent the mean ± SEM (*n* = 12–18 replicates of three independent experiments). Student unpaired *t* test, *P < 0.05; ***P < 0.001. *O* oligomycin, *F* FCCP, *R/A* rotenone/antimycin A

The same experiments were conducted to characterize wtTau and P301L cells (Fig. 2). No significant difference in basal respiration, ATP turnover, and glycolytic reserve was found between the two cell lines (Fig. 2a–c). However, wtTau cells had higher maximal respiration and spare respiratory capacity than P301L-transfected cells, indicating that mutant cells have some level of metabolic impairment, especially with regard to their mitochondrial reserve capacity (Fig. 2c). ATP levels were also significantly reduced in P301L cells (−27 % compared to wtTau cells) (Fig. 2d), which was paralleled by a depolarization of the mitochondrial membrane potential (decreased MMP, −10 % compared to wtTau cells) (Fig. 2e).

Taken together, these results confirm that APP/Aβ and hyperphosphorylated tau exhibit a negative impact on mitochondrial function leading to mitochondrial respiration deficiency and diminished ATP outcome. Since different bioenergetic parameters are impaired between APP and P301L cells, Aβ and abnormal tau appear to exert a different degree of toxicity on mitochondrial function.

### Sex steroid hormones distinctively increase mitochondrial bioenergetics in APP/Aβ and tau-overexpressing cells

**Fig. 3:**
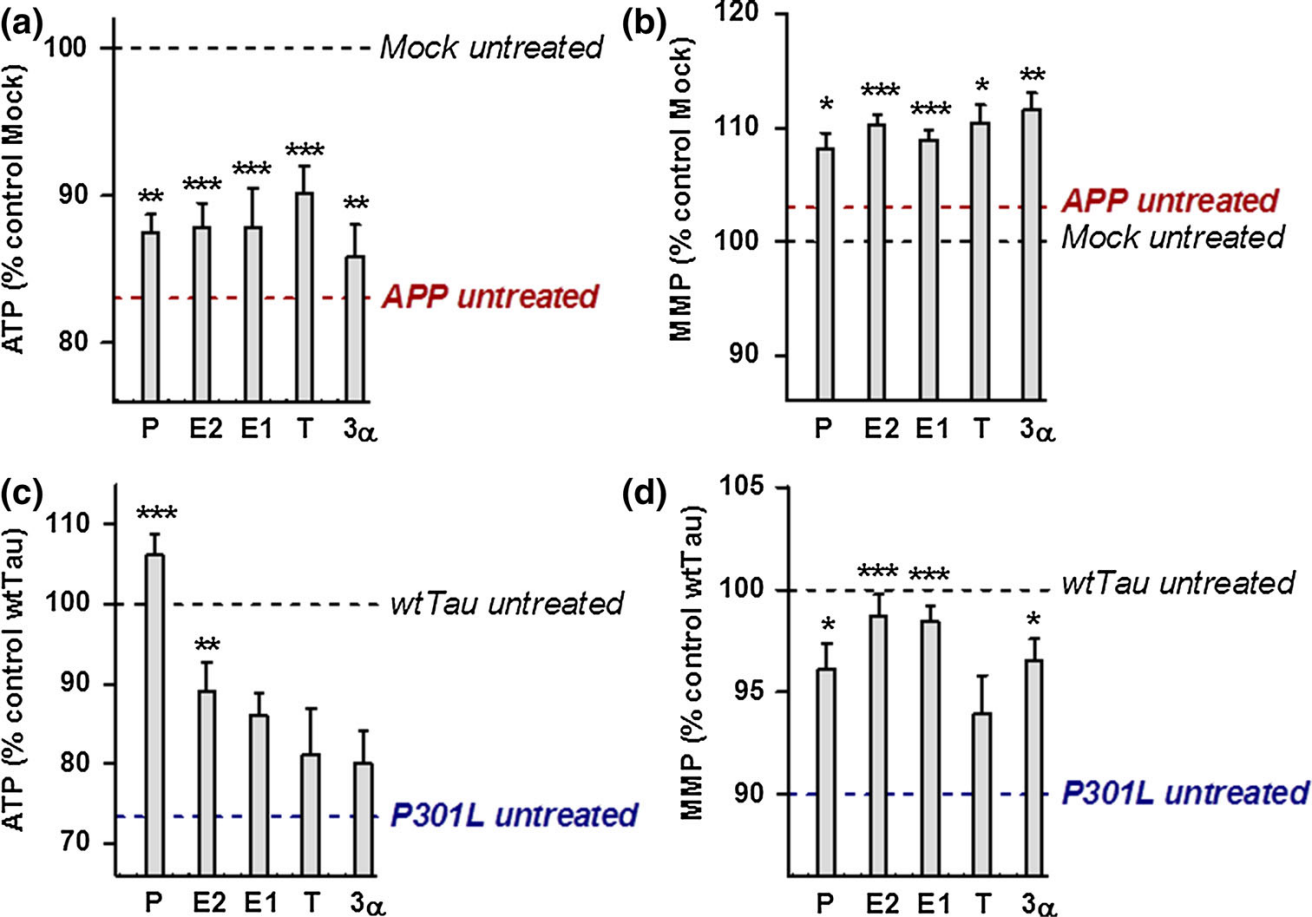
Neurosteroids increase ATP level and MMP in APP and P301L cells. ATP levels and MMP were measured after neurosteroid treatment for 24 h at a concentration of 100 nM in APP cells (**a**–**b**) and P301L cells (**c**–**d**), respectively. Values represent the mean ± SEM (*n* = 12–18 replicates of three independent experiments) and were normalized to 100 % of untreated Mock cells (**a**–**b**) or untreated wtTau cells (**c**–**d**). The values for untreated APP (**a**–**b**) and P301L cells (**c**–**d**) were also indicated by a *dashed line*. One-way ANOVA and post hoc Dunnett’s multiple comparison test versus untreated Mock or wtTau, *P < 0.05; **P < 0.01; ***P < 0.001. *P* progesterone, *E2* estradiol, *E1* estrone, *T* testosterone, *3α* 3α-androstanediol

**Fig. 4:**
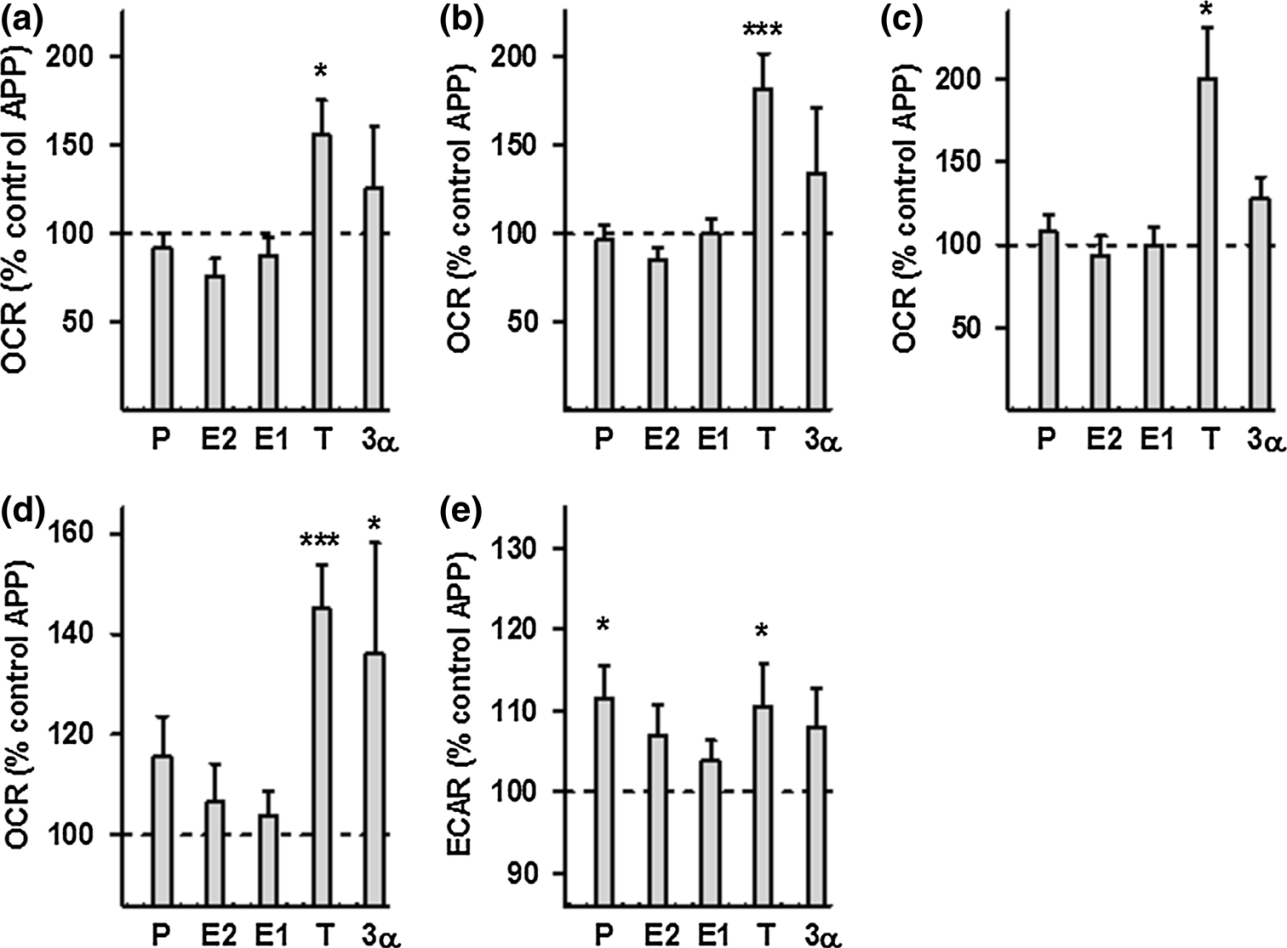
Effects of neurosteroids on bioenergetic parameters in APP cells. **a** Basal respiration, **b** ATP turnover, **c** maximal respiration, **d** spare respiratory capacity, and **e** glycolytic reserve were measured after neurosteroid treatment for 24 h at a concentration of 100 nM in APP cells, using a XF24 Analyzer (Seahorse Bioscience). Values represent the mean ± SEM (*n* = 8–10 replicates) and were normalized to 100 % of the control group (untreated APP cells, *dashed line*). One-way ANOVA and post hoc Dunnett’s multiple comparison test versus control, *P < 0.05; ***P < 0.001. *P* progesterone, *E2* estradiol, *E1* estrone, *T* testosterone, *3α* 3α-androstanediol

**Fig. 5:**
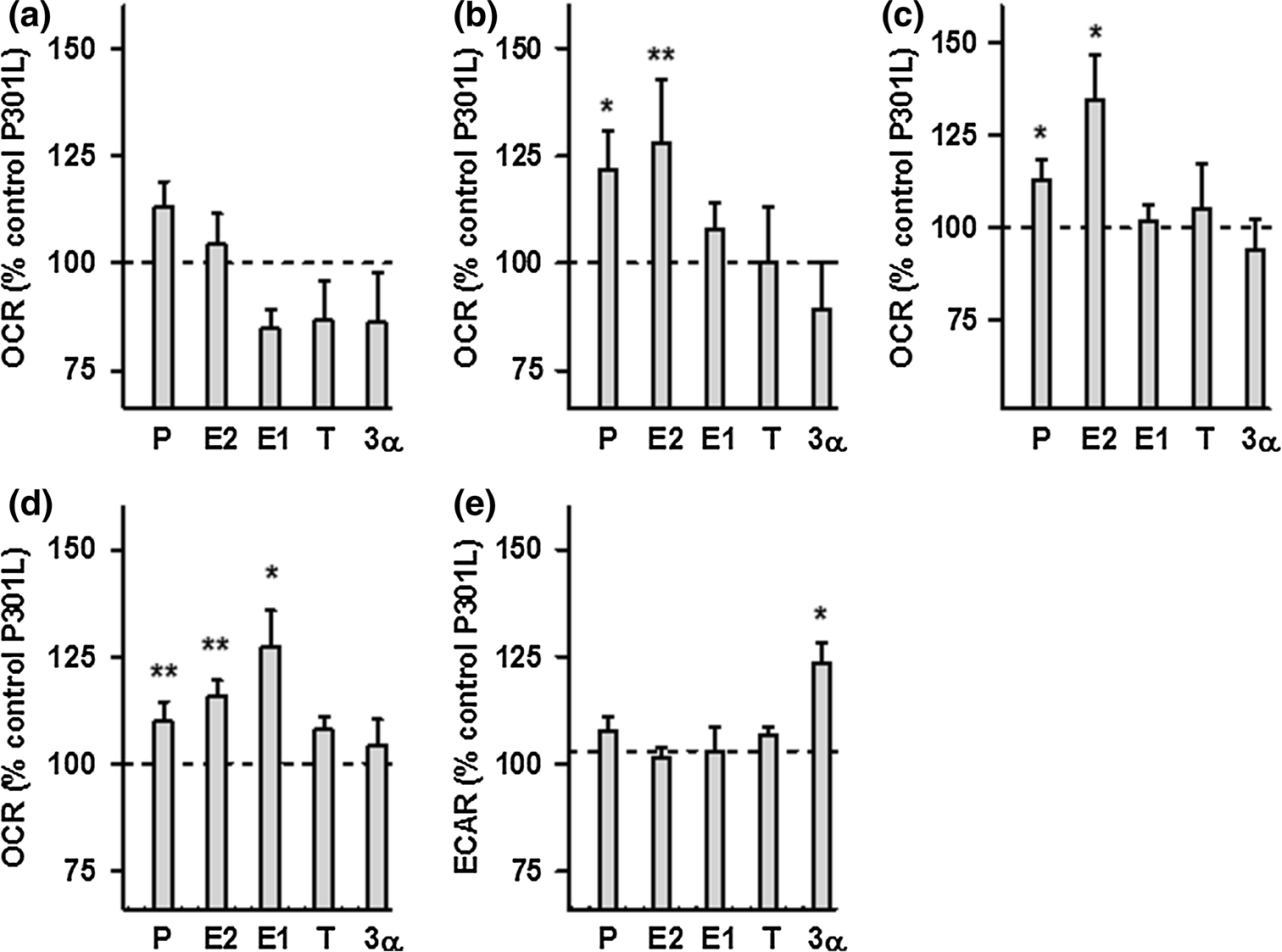
Effects of neurosteroids on bioenergetic parameters in P301L cells. **a** Basal respiration, **b** ATP turnover, **c** maximal respiration, **d** spare respiratory capacity, and **e** glycolytic reserve were measured after neurosteroid treatment for 24 h at a concentration of 100 nM in P301L cells, using a XF24 Analyzer (Seahorse Bioscience). Values represent the mean ± SEM (*n* = 8–10 replicates) and were normalized to 100 % of the control group (untreated P301L cells, dashed line). One-way ANOVA and post hoc Dunnett’s multiple comparison test versus control, *P < 0.05; ***P < 0.001. *P* progesterone, *E2* estradiol, *E1* estrone, *T* testosterone, *3α* 3α-androstanediol

To determine whether treatment with neurosteroids can improve mitochondrial function in AD cell culture models, ATP levels and MMP were analyzed in APP/Aβ and tau-overexpressing cells after 24 h of treatment (concentration 100 nM) with a range of steroids: progesterone (P), estradiol (E2), estrone (E1), testosterone (T), and 3α-androstanediol (3α) (Fig. 3). In undifferentiated APP cells, all steroids tested by us were able to significantly increase ATP levels as well as MMP (Fig. 3a, b), while in the differentiated state of APP cells only the male sex hormone testosterone was effective (Suppl. Figure 1). In contrast, in P301L cells, the female sex hormones progesterone and estradiol induced a significant increase in ATP levels in the undifferentiated (Fig. 3c) as well as differentiated state (Suppl. Figure 1). Progesterone was particularly effective since ATP levels in P301L cells were even higher (+6 %) compared to those of the untreated wtTau cells. In addition, all the tested steroids, except for testosterone, significantly increased MMP in P301L cells (Fig. 3d), with estrogens (E2 and E1) having the highest effect (+8 % compared to untreated P301L cells). To characterize the bioenergetic modulating profile of sex steroids on APP/Aβ and tau-overexpressing cells, measurements with a Seahorse Bioscience XF24 analyzer were performed after 24 h of treatments (at a 100 nM concentration) (Figs. 4, 5). In APP/Aβ overexpressing cells, only the testosterone treated group exhibited a higher basal respiration (Fig. 4a), ATP turnover (Fig. 4b), maximal respiration (Fig. 4c), spare respiratory capacity (Fig. 4d), and glycolytic reserve (Fig. 4e) compared to the untreated control group. In addition, 3α-androstanediol induced an improvement in the spare respiratory capacity (Fig. 4d) and progesterone significantly increased glycolytic reserve (Fig. 4e). These data suggest that especially testosterone, the main male sex hormone, exhibits a beneficial impact on mitochondrial malfunction in AD cells that are modeling the Aβ pathology.

Regarding the bioenergetic modulating profile of sex steroids on P301L cells, no significant changes were present in basal respiration (Fig. 5a). Nevertheless, the two main female sex hormones, progesterone and estradiol, significantly enhanced ATP turnover (Fig. 5b), maximal respiration (Fig. 5c), as well as spare respiratory capacity (Fig. 5d) compared to the untreated P301L cells. Estrone (E1), another estrogen, was also able to significantly increase spare respiratory capacity (Fig. 5d), and 3α-androstanediol was the only steroid able to grow the glycolytic reserve in P301L cells (Fig. 5e). Together, these data suggest that mainly female sex steroid hormones, progesterone and estradiol/estrone, improve mitochondrial bioenergetics in cells modeling tau pathology.

**Fig. 6:**
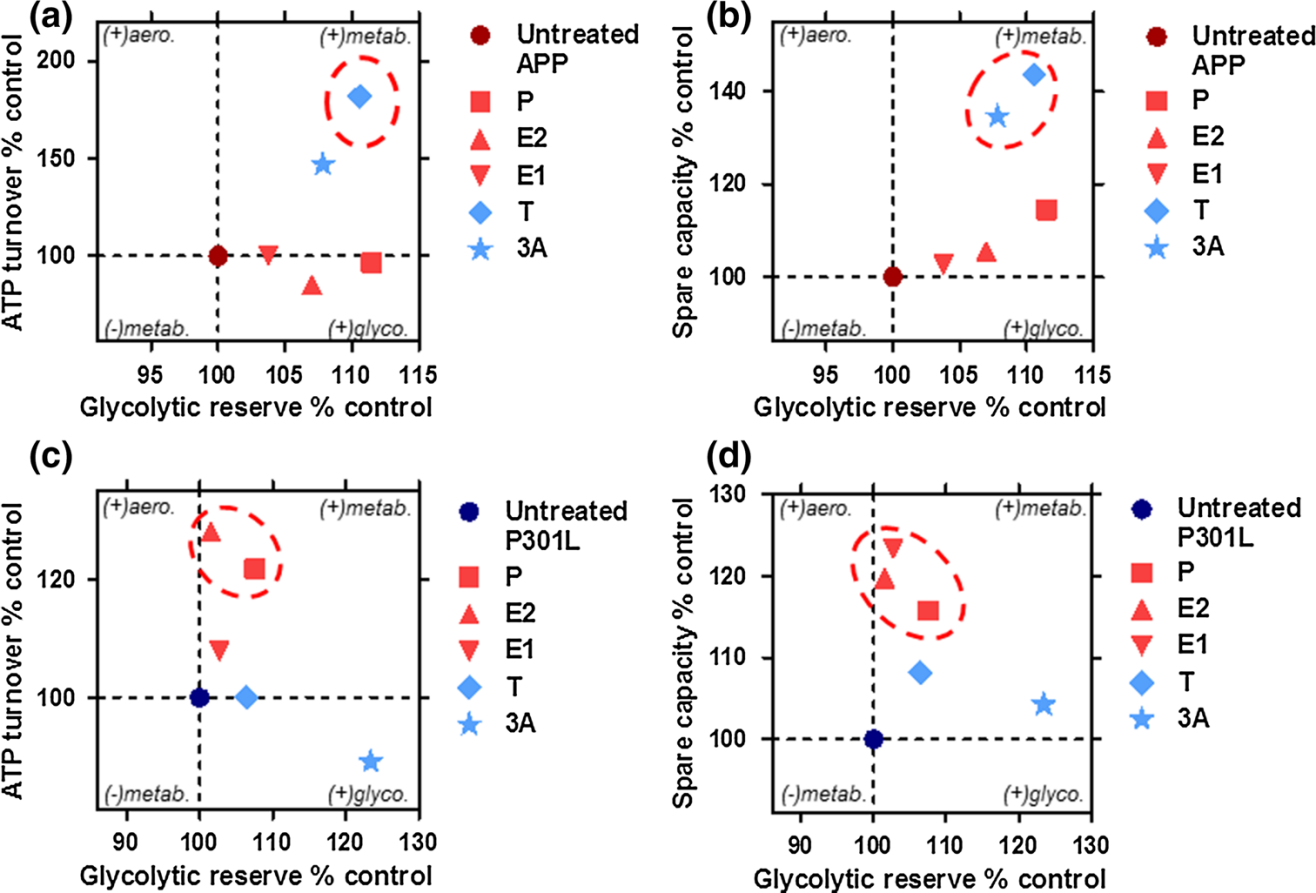
Neurosteroids differentially regulate the bioenergetic profile in APP/Aβ and abnormal tau-overexpressing cells. **a**–**b** Characterization of bioenergetic profiles of APP cells after neurosteroid treatment along two axes. Degree of **a** ATP turnover or **b** spare respiratory capacity is shown (in ordinate) in function of glycolytic reserve (in abscissa). The same parameters are displayed for P301L cells (**c**–**d**), respectively. Values represent the mean of each group normalized to the control group (untreated APP or P301L cells = 100 %). Significant changes upon respiratory parameters are highlighted by *dashed circles*. *P* progesterone, *E2* estradiol, *E1* estrone, *T* testosterone, *3α* 3α-androstanediol, *aero.* aerobic, *metab.* metabolic, *glyco.* glycolytic

A full analysis of preference for oxidative phosphorylation as indicated by percent of OCR dedicated to ATP turnover (Fig. 6a, c) or spare respiratory capacity (Fig. 6b, d) versus the use of glycolytic reserves was performed in both APP/Aβ and tau-overexpressing cells. A tendency for higher or lower metabolic activity is displayed on a second axis. Overall, APP cells were switched to a metabolically more active state after treatment with androgenic compounds (testosterone and 3α-androstanediol), with a tendency to increase both glycolytic reserve (ECAR) and ATP turnover/spare respiratory capacity (OCR) (Fig. 6a–b). In P301L cells, ATP turnover and spare respiratory capacity were enhanced by progesterone and estrogenic compounds (estradiol and estrone), leading to a more aerobic state (Fig. 6c–d).

Taken together, these results indicate that distinct sex steroid hormones are able to improve mitochondrial bioenergetics in APP/Aβ and tau-overexpressing cells by increasing ATP levels, MMP, and mitochondrial respiration which contribute to the alleviation of mitochondrial deficits observed in those cell lines. In this regard, the main players in the group seem to be progesterone, estradiol (E2), and testosterone.

Protective effects of neurosteroids against the oxidative stress-induced drop of ATP levels.

Since enhanced and unopposed metabolism-driven oxidative stress has a major role in age-related diseases including AD, we further wanted to assess whether sex hormone-related neurosteroids also exert a protective effect against oxidative stress. Therefore, we investigated ATP levels in APP/Aβ- and mutant tau-overexpressing cells after a 24 h pre-treatment with progesterone, estradiol, and testosterone followed by a 3 h treatment with hydrogen peroxide (H_2_O_2_). Because the two cell lines express different thresholds for vulnerability to oxidative stress, we used the respective concentration of H_2_O_2_ that induced a slight drop of about 10–15 % in ATP levels, namely 250 μM for APP cells and 500 μM for P301L cells (data not shown).

**Fig. 7:**
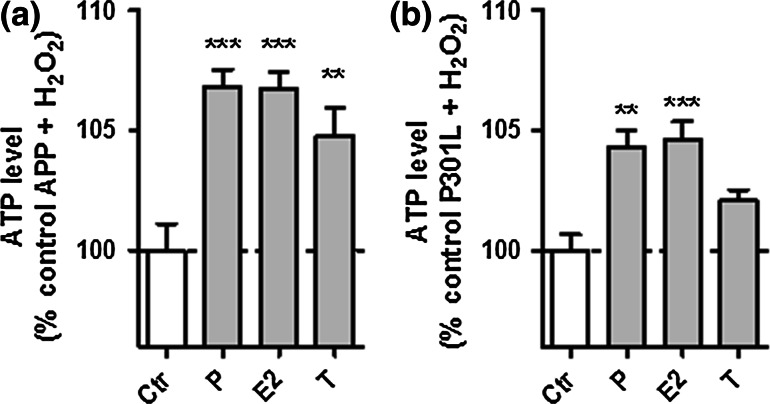
Neurosteroids are protective against the H2O2-induced drop of ATP levels in APP and P301L cells. **a** APP cells and **b** P301L cells were pre-treated 24 h with progesterone (*P*), estradiol (*E2*), and testosterone (*T*) and then stressed with **a** 250 μM H2O2 (APP cells) or **b** 500 μM H2O2 (P301L cells). Values represent the mean ± SEM (*n* = 12–18 replicates of three independent experiments) and were normalized to 100 % of APP cells (**a**) and P301L cells (**b**) not pre-treated with our selection of neurosteroids (*Ctr* control). One-way ANOVA and post hoc Dunnett’s multiple comparison test versus control APP or P301L, *P < 0.05; **P < 0.01; ***P < 0.001

Similar to the unstressed situation, all three neurosteroids were able to significantly attenuate the H_2_O_2_-induced drop in ATP levels in APP cells (Fig. 7a), while progesterone and estradiol were again selectively effective in P301L cells (Fig. 7b).

Together, these results indicate that sex steroid hormones may exert a distinct protective effect in APP/Aβ and mutant tau-overexpressing cells by increasing ATP levels when cells are exposed to oxidative stress condition.

## Discussion

In this study, we distinguished the effects of several neurosteroids on ATP synthesis, the MMP, mitochondrial respiration, and glycolysis in two cellular AD models. Our key findings were that (1) APP/Aβ and mutant tau-overexpressing cells present distinct bioenergetic impairments, with APP/Aβ having the strongest deleterious effect on mitochondrial function and (2) the male steroid hormone, testosterone, was more efficient in alleviating mitochondrial deficits in a model of AD-related amyloidopathy, whereas the female steroid hormones, progesterone and estrogen, were more efficient in increasing bioenergetic outcomes in a model of AD-related tauopathies. In addition, this protective pattern was evident under physiological as well as oxidative stress conditions.

Remarkably, bioenergetic profiles were differentially impacted in APP/Aβ-overexpressing cells and abnormally hyperphosphorylated tau-overexpressing cells. Only the maximal respiration and spare respiratory capacity were reduced in P301L cells, while APP cells presented, in addition, a decrease in basal respiration, ATP turnover, and glycolytic reserve. A drop in ATP levels was observed in both cell lines as well as a decreased MMP in P301L cells. Interestingly, APP cells presented with a slightly hyperpolarized mitochondrial membrane compared to the Mock control cells. This characteristic was previously observed in PC12 cells overexpressing APP bearing the Swedish mutation (APP^sw^), another cellular model mimicking Aβ pathology [33]. The authors hypothesized that this hyperpolarization may be due to increased nitric oxide levels present in this cell line when Aβ production is enhanced. Of note, Aβ secretion was similar in the wild-type APP-overexpressing human SH-SY5Y cells used in the present study and in APP^sw^-overexpressing PC12 cells within the low nanomolar range [31, 33], whereas higher Aβ levels obviously lead to MMP depolarization [33].

The different bioenergetic output observed between APP/Aβ and abnormal tau-overexpressing cells can be explained by the fact that Aβ and tau target mitochondria differentially [34]. Indeed, previous data from our group had shown that APP cells present a decreased mitochondrial complex IV activity [26], whereas complex I activity was impacted in P301L cells [27]. In line with, these findings are previous data from our group showing that a treatment of native SH-SY5Y cells with Aβ peptide decreased complex IV activity, which was coupled to a decrease in the respiratory control ratio [35]. Notably, deregulation of complex IV activity, and content, was previously observed in platelets, in fibroblasts, and in the brain of SAD patients already at early stages of the disease [8]. Moreover, we had shown that the Aβ peptide and abnormally hyperphosphorylated tau protein may act synergistically to trigger mitochondrial dysfunction in a triple transgenic mouse model of AD (^triple^AD) obtained after crossing P301L tau transgenic mice with APP^sw^PS2 double-transgenic mice [36]. The investigation of oxidative phosphorylation (OXPHOS) activity had revealed that deregulation of complex I activity was related to tau, whereas deregulation of complex IV activity was dependent on Aβ.

Thus, on the one hand, the lower complex I activity observed in P301L cells may lead to a decreased ability to reach maximal respiration, which reduces the spare respiratory capacity of the cells. On the other hand, the reduced complex IV activity, which is directly involved in oxygen consumption, may decrease additional respiratory parameters, including ATP turnover in APP cells.

Whether mitochondrial deficits are the causes or consequences of Aβ accumulation and tau hyperphosphorylation is still under debate. Recent reports indicate that mitochondrial dysfunction may represent the missing link between normal aging and SAD. As a genetic disorder, FAD clearly is a consequence of malfunctioning/mutated genes, while late-onset sporadic AD is more likely due to a gradual accumulation of age-related malfunction. Normal aging and AD are both marked by defects in brain metabolism and increased oxidative stress, albeit to varying degrees. Mitochondria are involved in these two phenomena by controlling cellular bioenergetics and redox homeostasis. Consequently, Swerdlow and colleagues placed mitochondria in the center of neurodegenerative processes in the “Alzheimer mitochondrial cascade hypothesis” [15]. This hypothesis postulates that the decline of mitochondrial function observed during aging, namely the decrease in energy production and the increase in ROS production and oxidative stress, eventually surpasses a threshold, thereby giving rise to the amyloidogenic pathway leading to Aβ accumulation. This triggers a vicious cycle in which Aβ may exacerbate mitochondrial deficits, creating other vicious cycles that also involve tau hyperphosphorylation, and eventually lead to neurodegeneration and dementia [37, 38]. Thus, there exists a very complex, and yet not well understood, interaction between the mainly age-related early onset of mitochondrial deficits, Aβ accumulation, and tau hyperphosphorylation, emphasizing that interventions targeting mitochondrial deficits may serve as potential strategies for the prevention or treatment of age-related neurodegenerative disorders.

In our study, the treatment with sex steroid hormones was able to alleviate the bioenergetic impairments observed in APP and P301L cells in general. More specifically, the male hormone testosterone was able to enhance all the bioenergetic parameters that were impaired in APP/Aβ overexpressing cells, namely basal respiration, ATP turnover, maximal respiration, and cellular glycolysis as well as ATP levels specifically in differentiated APP cells, indicating that cell sensitivity towards this hormone may increase during differentiation. In contrast, treatment with female hormones improved maximal respiration and spare respiratory capacity, two bioenergetic parameters that were disturbed in P301L cells. ATP turnover is an indication of the coupling efficiency that is directly linked to ATP production in mitochondria. Bioenergetic profiling revealed that male and female steroid hormones were able to differentially increase ATP synthesis in APP and P301L cells, respectively. The spare respiratory capacity and glycolytic reserve provide an indication of the ability of a cell to respond to stress under conditions of increased energy demand [39]. The spare respiratory capacity was increased by estrogens and progesterone in P301L cells, whereas both parameters were enhanced after treatment with androgen in APP cells. Together, the data indicated that the cells were switched to a metabolically more active state, with a tendency to increase both ATP synthesis and metabolic reserves. In addition, male and female sex hormones were able to increase ATP levels after oxidative injury in APP cells, when only progesterone and estradiol showed a significant effect on this bioenergetic parameter in P301L cells. These data suggest that the neurosteroids belonging to the sex hormone family may not only exert neuroprotective effects against Aβ- or tau-related toxicity but also against oxidative stress, a key feature of brain aging.

The ability of neurosteroids to modulate cellular bioenergetics and redox homeostasis was the focus of a recent study by our group. In particular, we showed that neurosteroids, including the steroids that were investigated in the present study, were able to increase ATP levels and mitochondrial respiration in native SH-SY5Y neuroblastoma cells and mouse cortical neurons [19]. In parallel, they modulated redox homeostasis by increasing antioxidant activity, probably as a compensatory response to oxidative stress due to a slight enhancement in reactive oxygen species (ROS) levels resulting from the neurosteroid-induced boost in oxygen consumption [19]. Moreover, we showed that the effects we observed were, at least in part, mediated by steroid (progesterone, estrogen, and androgen) receptor activation since the inhibition of those receptors by specific antagonists shut down the effects of the corresponding steroid ligand on ATP production.

Steroid receptors are nuclear receptors involved in the regulation of gene expression. With regard to bioenergetics, estrogens have been shown to up-regulate genes encoding some of the electron transport chain components such as not only subunits of mitochondrial complex I (CI), cytochrome c oxidase (complex IV), and the F1 subunit of ATP synthase but also glucose transporter subunits, enzymes involved in the tricarboxylic acid cycle (TCA cycle) and glycolysis, leading to increased glucose utilization and mitochondrial respiration (reviewed in [18]). Of note, since the mitochondrial genome itself contains hormone responsive elements, it has been proposed that estradiol and testosterone can regulate energy production by inducing mitochondrial oxidative phosphorylation (OXPHOS) genes encoded in the mitochondrial DNA [40]. In a similar way, progesterone has been shown to increase complex IV and V (ATP synthase) expression, accompanied by enhanced mitochondrial respiratory activity [41]. With regard to the results obtained in the present study, we can speculate that the underlying mechanisms are similar and that the effects we observed are, at least in part, mediated by an increased expression of genes involved in OXPHOS and glycolysis. Further investigations will be needed to identify in detail which genes are concerned.

It is interesting to observe that sex steroid hormones present distinct mitochondrial improvements with regard to their bioenergetic profiles in the presence of Aβ-related or tau-related mitochondrial dysfunction. Estrogens and progesterone seem to confer beneficial effects on mitochondrial-related dysfunction preferentially in tau pathology, whereas testosterone was more efficient in alleviating mitochondrial deficits in APP/Aβ overexpressing cells. These findings may imply that women and men differentially respond to mitochondrial insults mediated by either Aβ or abnormal tau.

Epidemiological studies report a higher prevalence and incidence of AD in women even as well as a greater vulnerability to the disease, since they represent two thirds of SAD patients [42, 43]. In particular, AD pathology is more strongly associated with clinical dementia in female patients than in male [44]. Besides, women exhibit greater senile plaque deposition than men already at early stages of neurofibrillary tangle development [45] and show a higher vulnerability to oxidative damage [46]. These observations are also made in transgenic animal models. Indeed, in simple (Tg2576) [47], double (APP/PS1) [48], as well as in triple (3xTg-AD) transgenic mice [49], an increased Aβ load burden and plaque number was found in the female brain compared to age-matched male mouse brain. These differences were even more striking in old females after the age of 11 months when the estrous cycle became irregular or when animals were ovariectomized (OVX). In these females, a significant enhancement of Aβ load in important brain regions like the hippocampus was observed [49, 50]. In addition, in female 3xTgAD mice, mitochondrial bioenergetic deficits and oxidative stress, which occur before the onset of AD pathology, were exacerbated in OVX females compared to sham OVX animals [50, 51]. A treatment with female sex hormones (estradiol or progesterone) was able to reduce Aβ load and NFT formation and also rescue mitochondrial deficits in OVX 3xTgAD mice [50, 52]. Interestingly, in a study using demasculinized 3xTgAD males, these animals exhibited a significant increase in brain Aβ load compared to normal males, while in defeminized female mice, Aβ accumulation was comparable to that of males in some brain regions [49].

Taken together, these findings highlight the important role of sex hormones in the development of AD in both male and females.

Indeed, the sudden drop in estrogen levels in women after menopause has been proposed to be one risk factor in AD. Indeed, estradiol is the major product of estrogen biosynthesis, and it remains the most abundant estrogen in a woman’s pre-menopausal life. After menopause, women have estradiol levels comparable to men, and it is then that women become more susceptible to AD. Thus, the precipitous decline of estrogens during menopause may contribute to AD onset as well as a greater vulnerability to the disease in women [18]. Men, in contrast, present with a gradual reduction in testosterone over the life course eliminating approximately 2 % of circulating testosterone every year [53]. Interestingly, human and animal studies also suggest that androgen deprivation represents a risk factor for AD pathogenesis [54–56]. Notably, in a triple transgenic mouse model of AD (3xTgAD), it has been shown that orchiectomized males presented with increased Aβ accumulation in the brain, coupled with impaired cognitive performances compared to sham-operated mice [57]. Treatment with androgens significantly attenuated the increase in AD pathology [55, 57]. Further studies have indicated that advancing age in men enhances tau hyperphosphorylation consistent with AD pathology [58]. These findings suggest that the influence of steroids on tau remains a promising, yet underexplored research avenue in AD. Of note, no sex predilection has been identified in patients with FTDP-17, a disease characterized by a strictly tau-dependent pathology [59, 60]. However, in a study using mice bearing the P301S tau mutation, a greater tau accumulation was evident in different brain region of males already at an age of 7 months, as well as a more apparent mitochondria proteome deregulation in male P301S mitochondria [61].

Together, these findings suggest that the relationship between hormonal loss and the risk to develop AD may be preferentially linked to Aβ pathology in females and tau pathology in males.

A few studies have focused on the impact of steroids on abnormal tau. Liu and colleagues discovered that protein kinase A may initiate phosphorylation of tau, and estradiol treatment of human embryonic kidney cells attenuated protein kinase A activity as well as reduced tau phosphorylation [62]. Estradiol also exhibited a rescue of aberrant tau in primary rat cortical neurons and SH-SY5Y neuroblastoma cells [63]. Additional studies showed that both estrogen and progesterone were able to modulate activities of kinases and phosphatases involved in the regulation of tau phosphorylation, possibly by modulating the glycogen synthase kinase (GSK) pathway [54]. Specifically, estrogen appeared to reduce GSK-3β activity while progesterone decreased the expression of both GSK-3β and tau [54, 63]. Regarding AD-linked Aβ pathology, studies have focused on the impact of estrogen on the deposition and clearance of Aβ [64, 65]. Both estrone and estradiol decreased polymerization and stabilization of Aβ [65, 66]. Other studies indicate that deficiencies in estrogen-related steroids can exacerbate Aβ plaques in AD mouse models and that treatment with estradiol was able to reduce Aβ burden, possibly via stimulating the non-amyloidogenic pathway of APP processing [64, 67]. The effects of progesterone on Aβ deposition and clearance are less investigated, but a recent study showed that progesterone and estradiol encouraged an increase in the expression of Aβ clearance factors both in vitro and in vivo [68].

In the present study, testosterone was shown to ameliorate the effects of mitochondrial dysfunction caused by APP/Aβ but not abnormal tau. In our previous study investigating neurosteroid effects on bioenergetics under physiological conditions, the testosterone metabolite, 3α-androstanediol, presented an effect similar to its precursor and was able to increase MMP, ATP levels, and mitochondrial respiration in untransfected neuroblastoma cells and primary cortical cells [19]. Here, 3α-androstanediol was less efficient in alleviating bioenergetic deficits in APP cells, suggesting a distinct mode of action compared to testosterone. Neuroprotective effects of testosterone on hyperphosphorylated tau are less well characterized in the literature. Testosterone appears to prevent tau hyperphosphorylation in an in vivo model of heat shock induced phosphorylation through inhibition of GSK-3β signaling [58]. Interestingly, Rosario and colleagues [56] revealed reduced abnormal tau accumulation in gonadectomized male 3xTgAD mice treated with testosterone or estradiol but not the testosterone metabolite dihydrotestosterone (DHT). This implies that testosterone may exert indirect effects on tau hyperphosphorylation via its conversion to estradiol by the enzyme aromatase and by acting on estrogen receptors. In the same model, testosterone and DHT were able to decrease Aβ deposits with a higher efficiency than estradiol, suggesting an androgen receptor-dependent mechanism. Overk and colleagues (2013) [69] examined basal levels of serum and brain testosterone in male 3xTgAD mice and found that testosterone levels rise with disease progression. This increase in testosterone in aged male 3xTgAD mice was correlated with a reduced Aβ plaque pathology. This suggests that testosterone may have some neuroprotective benefits on the AD disease course, but that testosterone administration is associated more with a lower Aβ protein burden rather than abnormal tau protein. In fact, testosterone has been shown to alter processing of amyloid precursor protein and enhances expression of neprilysin, an Aβ-degrading enzyme [70].

The decrease in sex steroid hormones was proposed to be one risk factor of AD in both men and women. However, there is little information concerning changes of steroid levels in the human brain during aging and under dementia conditions. Steroid hormones originating from the endocrine glands can freely pass through the blood–brain barrier and act on nervous tissues. Since steroids can also be synthesized within the nervous system, changes in blood levels of steroids with increasing age do not necessarily reflect changes in brain levels. Schumacher and colleagues quantified levels of different neurosteroids in various brain regions of aged AD patients and aged non-demented controls [24]. They showed a general trend towards lower neurosteroid levels in AD patients. Additionally, neurosteroid levels were negatively correlated with Aβ and abnormal tau in some brain regions, suggesting a link between neurosteroid homeostasis and AD pathogenesis. However, large studies investigating systematically gender differences with respect to Aβ and/or tau pathology in post-mortem brains from AD patients are still missing. Since the two hallmarks are present in AD brains and are both involved in the pathophysiology of the disease, a strict dichotomy between Aβ- and tau-related deficit in females and males, respectively, is not easy and requires a more thoughtful investigation. In addition, one needs to consider that, even if the brain and the peripheral levels of steroids are not directly correlated in all cases, circulating sex hormones contribute to the brain pool of steroids, due to their ability to cross the blood–brain barrier. Indeed, evidence is provided that a decrease in circulating steroid hormones, during aging or after ovar-/gonadectomy, is accompanied by a decrease in brain levels of these steroids [55, 71, 72]. In this context, different concepts of hormonal replacement therapies for women and men are currently discussed with regard to the use of estradiol and testosterone, respectively, to assess the effect of sex hormones in decreasing the risk of dementia, including AD [73, 74]. Longitudinal proof-of-concept studies are now needed to identify an optimal window of opportunity to optimize treatment efficacy as well as safety.

Our experiments revealed a rescue of metabolic dysfunction in models of AD-linked tauopathies and amyloidopathies with neurosteroids belonging to the sex hormone family. Taking into account the data available in the literature and in our previous study, this rescue may possibly occur at two levels: (1) neurosteroids may directly boost mitochondrial function via gene regulation and (2) neurosteroids may decrease Aβ accumulation and NFT formation, thereby alleviating mitochondrial impairments induced by Aβ and tau. An interaction between these mechanisms cannot be excluded.

Given the complex interaction that seems to link mitochondrial function, gender, and the pathophysiology of AD, our findings have shown that sex steroid hormones may represent interesting therapeutic tools to counteract bioenergetic deficits in AD. Thus, experiments dissecting the mechanistic pathways of neurosteroid function underlying the gender differences in AD are interesting research paths for the better understanding of how neurosteroids impact mitochondrial function in AD. Ultimately, our research will potentially open new avenues for the development of gender-specific therapeutic strategies in AD.

## Electronic supplementary material

Suppl. Figure 1: Neurosteroids increase ATP level in differentiated APP and P301L cells. ATP levels were measured after neurosteroid treatment for 24 h at a concentration of 100 nM in APP cells **(a)** and P301L cells **(b)** respectively. Values represent the mean ± SEM (n = 12-18 replicates of three independent experiments) and were normalized to 100 % of untreated APP cells **(a)** or untreated P301L cells **(b).** One-way ANOVA and post hoc Dunnett’s multiple comparison test versus untreated (Ctr = control) APP or P301L cells, *P < 0.05; **P < 0.01; ***P < 0.001. P; progesterone, E2; estradiol, T; testosterone. (TIFF 577 kb**)**

## Abbreviations

3α-A: 3α-androstanediol
Aβ: Amyloid-β peptide
AD: Alzheimer’s disease
APP: Amyloid-β precursor protein
DMSO: Dimethylsulfoxide
E1: Estrone
E2: 17β-estradiol
ECAR: Extracellular acidification rate
ETC: Electron transport chain
MMP: Mitochondrial membrane potential
OCR: Oxygen consumption rate
OXPHOS: Oxidative phosphorylation
P: Progesterone
P301L: Tau mutation
PD: Parkinson’s disease
ROS: Reactive oxygen species
T: Testosterone
wtTau: Wild-type tau
