# Supplementary figures and images for: Sex hormone-related neurosteroids differentially rescue bioenergetic deficits induced by amyloid-β or hyperphosphorylated tau protein
Source: Cell Mol Life Sci. 2015 Jul 22;73(1):201–15. doi: 10.1007/s00018-015-1988-x (PMC4700074; doi:10.1007/s00018-015-1988-x)

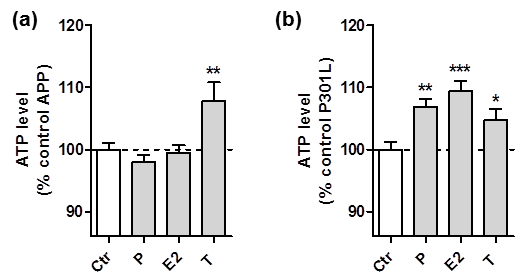

Supplement: Supplementary file 1 — Suppl. Figure 1: Neurosteroids increase ATP level in differentiated APP and P301L cells. ATP levels were measured after neurosteroid treatment for 24 h at a concentration of 100 nM in APP cells (a) and P301L cells (b) respectively. Values represent the mean ± SEM (n = 12-18 replicates of three independent experiments) and were normalized to 100 % of untreated APP cells (a) or untreated P301L cells (b). One-way ANOVA and post hoc Dunnett’s multiple comparison test versus untreated (Ctr = control) APP or P301L cells, *P < 0.05; **P < 0.01; ***P < 0.001. P; progesterone, E2; estradiol, T; testosterone. (TIFF 577 kb) [file 18_2015_1988_MOESM1_ESM.tif]
